# Some Abnormalities and Complications of the Pregnant State, with Report of Cases1Read before the Buffalo Academy of Medicine, May 23, 1910.

**Published:** 1910-12

**Authors:** L. G. Hanley

**Affiliations:** Buffalo, N. Y.; Gynecologist to Buffalo Hospital Sisters of Charity; Chief Obstetrician of St. Mary’s Infant Asylum and Maternity Hospital; to Surgeon Providence Retreat; Consulting Obstetrician Erie County Hospital; Gynecologist to the Emergency Hospital; Consulting Surgeon to the Infant Home Lady of Victory; Surgeon to the New York Central Railroad Co.; former Clinical Professor of Obstetrics University of Buffalo, and Professor of Obstetrics, Niagara University; 428 Porter Avenue


### BUFFALO MEDICAL JOURNAL

VOL. LXVI. DECEMBER, 1910. NO. 5

### ORIGINAL COMMUNICATIONS

### Some Abnormalities and Complications of the Pregnant State, with Report of Cases[1]

#### Including Ectopic Gestation, Fibroid Tumors, Appendicitis, Obstruction of the Bowel and Tuberculosis

BY L. G. HANLEY, M.D., LL.D.,
Buffalo, N. Y.

Gynecologist to Buffalo Hospital Sisters of Charity; Chief Obstetrician of St. Mary's Infant Asylum and Maternity Hospital; to Surgeon Providence Retreat; Consulting Obstetrician Erie County Hospital; Gynecologist to the Emergency Hospital; Consulting Surgeon to the Infant Home Lady of Victory; Surgeon to the New York Central Railroad Co.; former Clinical Professor of Obstetrics University of Buffalo, and Professor of Obstetrics, Niagara University.

THE term ectopic is derived from the Greek *ektoños* which means out of the normal place. The proper habitat for the fecundated ovum is the uterus, and when we use the term ectopic gestation we mean that the product of conception or pregnancy is extrauterine, or perhaps in a rudimentary horn of a bicornate uterus. Anything that will interfere with the transit of the ovum, whether it be congenital malformation, or some other pathological cause, will produce the condition known as ectopic pregnancy.

The uterus is the home of the fetus, but frequently this product of conception in its youth does not go home but likes to wander from its own fireside, perhaps due to the obnoxious presence and results of the gonococcus, or the staphylococcus, or to the bacillus coli, that have spoiled its environment and surroundings, or perhaps it is due to "wanderlust." Whenever this happens it may possibly outlive its hobo life of wandering, go to full term and come home, like the prodigal, in an emaciated and starved condition; but the chances are as records show, that it will be cast by the wayside and die of starvation, its means of sustenance, that is, its blood supply, being cut off.

It matters not whether it leads tubal, ovarian, or abdominal existence, the treatment is the same and it is bound to receive surgical chastisement. Tubal is by far the most common and it is a question with some authors if all cases of ectopic pregnancy are not primarily tubal. If a diagnosis can be made before rupture, which is possible in nearly every case, then the dan-

1. Read before the Buffalo Academy of Medicine, May 23, 1910.

ger is not so great as when rupture occurs. When rupture does occur then it is a question of "maximi momente" to open the abdominal cavity quickly and make a rapid operation.

The question of waiting after rupture has taken place, and the patient in great shock, the chances being that the shock may become still greater, has never appealed to me, for there is no telling when the plug in the bleeding aperture may break, hemorrhage start, and prove that procrastination was the thief that snatched the patient. We have seen rupture in ectopic cases where there was only little blood in the abdominal cavity, yet the patient was in extremis. One might say that here was a case where we could wait with propriety. I have seen cases where there was a great amount of blood in the abdominal cavity and the patient not in extreme shock. If that small amount of blood would produce a condition of extreme shock in one individual, what would a greater amount of blood do to the same individual? Each case is a problem in itself; and where certain individuals can stand only the effects of slight hemorrhages, others may withstand many times more without bad effect. There are many cases of extrauterine pregnancy that rupture and are absorbed, the patient experiencing very little inconvenience, and which have been discovered at a later period when the abdominal cavity was opened for some other pathological condition.

It is very seldom that extrauterine pregnancy goes to term, the early death of the fetus being due to its lack of nourishment. If there should be continuous hemorrhages a circumscribed hematoma may form in the pelvic cavity, which may become infected producing pelvic abscess with all its complications, dangers, peritonitis, suppuration and so forth. If there are repeated hemorrhages because the ovum is not wholly extruded, or not wholly absorbed, it may become infected, remain for a great length of time as an organised clot. This is the kind that can be operated with safety through the posterior culdesac. The same may be said when the tube ruptures downward between the folds of the broad ligament. It may be entirely extraperitoneal, and if of long standing may be opened from below.

"To know a subject thoroughly perhaps is not permitted unto erring mortals." But the symptoms of nearly all cases have something in common that lead to a very marked-like diagnosis. In the beginning it corresponds to normal pregnancy. Whether before rupture, after rupture, at time of rupture, and after peritonitis and suppuration have set in, there generally is a history of a missed period of menstruation, vaginal discharge, which may be scant in appearance whether this occurs shortly after a missed period, or after several weeks, and if the decidual membrane is found a diagnosis is certain. With the above symptoms if the woman is seized suddenly with violent pain, passing from one faint to another, pulse very rapid and feeble, with cold, clammy perspiration, face blanched and all symptoms of internal hemorrhage and shock, little temperature or sub-normal, a diagnosis is absolute.

All these symptoms may not manifest themselves, but only a cutting, grinding pain, with tenderness over the abdomen. The physician is frequently called because the woman thinks she is aborting, having missed a period and is passing shreds, blood and membrane. Cases exist in which there are no classic symptoms except the missed period and in which it is impossible to discover any swelling in either tube, even though the person thinks she is pregnant. She goes on for another month, she menstruates but says it does not appear normal, she says it stops and begins again. Now, by examining the discharge we may be able to discover the presence of the decidua, which proves conclusively the presence of an extrauterine fetation. If there be a missed period of menstruation, even absence of sharp pain, meteorism, accelerated pulse, and the previous history, coupled with a vaginal examination that reveals a bulging mass in the posterior culdesac, we are warranted in calling it extrauterine pregnancy and making an incision through the median line to confirm the diagnosis and cure the patient.

It is not my intention to enter too minutely into the etiology, symptoms, and physical signs, but to report some cases out of the ordinary from a record of seventy-eight patients that have been operated by me.

*Ectopic and pyosalpinx—Primipara.*

AUGUST 16, 1903.

Mrs. C., age 34. Previous history: has always suffered from dysmenorrhea; menstruated first at fifteen. Present history: has been married eight years, had gonorrhea contracted the year of her marriage. Menstruation stopped for six weeks. She said she had pain in her side, dizziness, and soreness, but did not faint and kept doing her work as usual. Wher first seen, temperature 99½, pulse 118, respirations 24, and complained of pain all through the lower abdomen. There is peritonitis; examination shows a thickened, hardened mass in the pelvis, bogy on the right side but hardened on the left. Diagnosis—"pelvic abscess." Patient was operated through median line and also from below. The right tube had ruptured, and there was considerable blood in the abdominal cavity. The left tube showed a pyosalpinx of long standing. Each of these were removed—

both the tube on the right and the pyosalpinx on the left,—and a vaginal drain put in. Patient died of septic peritonitis August 22, 1903.

May 4, 1906.

Mrs. F., age 32; number of children, 2; previous history indefinite. Woman was a Slav and spoke no English. Only history was "sick in belly." Examination showed a swelling in the region of the right tube, tender, with some bulging in the vault of the vagina. Operation. Found right tube had ruptured and a large hematoma of long standing which, with a fetus 1½ in. long, was removed. Bleeding very slight. Left tube apparently healthy. Recovery uneventful. May 17, 1908, she returned to the hospital suffering from pain in the left side, and presenting all symptoms of ectopic. Operation revealed a condition similar to that which existed two years previously. There was a rupture of the left tube, quantities of blood in the abdominal cavity but no fetus was found. She left the hospital in three weeks.

September 20, 1904.

Mrs. C., age 30, mother of two children, one a baby 11 months old; has had no abortion or infection, and has always been healthy. Pregnant six weeks, taken suddenly with sharp pain in side, fainting, and was compelled to go to bed; was treated by a physician for cramps; morphine ¼ grain was given. I saw her 24 hours after seizure; found a mass in the region of the right tube, pulse 128; slight tenderness and extension over the abdomen; my visit was at nine o'clock in the evening; operated at eleven. Abdomen contained about two quarts of blood. Patient left the hospital in three weeks. Subsequent history; patient became pregnant one month after operation, and was delivered by my assistant at that time of a full term baby, which is still living. Mother experienced no inconvenience from her operation, pregnancy, or labor following. The woman did not know that she was pregnant, had just weaned her baby and had not menstruated. She has had no infection and has been a busy woman since she was married.

March 7, 1910.

Mrs. C., age 31. Number of children five. Number of miscarriages five. Menstruated at eleven years. Has been irregular for the last two years. Menstruated last on December 7, 1909. Referred to me by Dr. A. Colton. She began flowing three weeks before entering the hospital; was brought here from a distance of eighty-six miles away. She was unconscious; she suffered greatly from pain and shock and flowed a pure red blood; no clots. Examination showed a bulging on the left side. Operation. About one quart of blood with clots in the abdominal cavity. Recovery uneventful. Fetus not found. Left hospital March 26, 1910.

*Primipara, no symptoms but a missed period.*

JULY 30, 1903.

Mrs. C., age 30; previous history negative; has never been pregnant; until two months before admission had always been regular. As this was a former patient of mine she sent for me to come to her home, saying that she had passed something from the vagina accompanied with pain. This woman complained of no early symptoms of pregnancy, said she expected she was pregnant and hoped she was, but had none of the symptoms that other women complain of,—not sick to her stomach, no headache or dizziness, no sensations in the breasts, in fact she said she felt perfectly well. I had her removed to the hospital and operated, finding a mass in the right tube that had not ruptured, containing a fetus of two months' growth, which I removed. Patient made a good recovery, and has since been pregnant twice, carrying each to full term.

*Ectopic—Pyosalpinx—Primipara—Peritonitis.*

MARCH 31, 1903.

Mrs. H., age 24, previous history of no importance. No children; always regular till last period. There was a right pyosalpinx; was taken sick one week before admitted to hospital. At that time she had pain in her right side, which had been continuous for one week; she had fainted several times; she was nearly in a state of collapse, temperature 99½, pulse 120, abdomen somewhat distended, and had all the appearances of a septic peritonitis—respiration 28. From the history of the case I decided that it was ectopic and prepared the patient for operation, and made an incision to the posterior culdesac where there was a great discharge of blood and clots. Opened then from above and found the left tube ruptured, which I removed. I also removed the pyosalpinx on the right. Patient died two days afterward from septic peritonitis.

OCTOBER 13, 1903.

Mrs. B. Primipara. Age 24. Previous history not important. Menstrual history normal. She has always been healthy till about six months ago when she had a fall, and has suffered since with pain in her back and with her menstruation. She would flow for two days, then stop for a week, when the flow would begin again; she passed some clots, shreds, and was continually getting weaker; she suffered from pain in her right side, and as she expressed it "was obliged to remain in bed half the time." She was anemic; headache; hemoglobin 30. Examination shows a bulging mass in the right side of the vaginal vault, soft; abdomen opened which showed the right tube had ruptured and a fetus of about two months growth was found among the clots. Right tube removed; left was normal; abdomen flushed with normal salt solution. Recovery uneventful.

*Left pyosalpinx with ectopic pregnancy.*

NOVEMBER 6, 1904.

Mrs. L., age 27; married three years. History prior to marriage not significant. Has had two miscarriages and has since been treated for ovarian trouble. Two weeks before entering hospital she experienced a sudden sharp pain in the abdomen, had a chill, fainted and was suffering from diarrhea. She gives a history of having missed no menstrual period; examination per vaginam shows a bulging mass on the left side with some thickening on the right. She was prepared for operation, and on opening the abdomen a left pyosalpinx was found, with pregnancy in the right tube, fetus protruding, and clots in the pelvic cavity. Fetus was two inches long. Patient made a good recovery. The history of this case shows that she had none of the presumptive and probable signs of pregnancy.

*Interstitial gestation—rupture during examination.*

SEPTEMBER 6, 1900.

Mrs. B., age 35. Previous history nothing out of the ordinary. Married five years, number of children two, abortions one. A year and a half before being admitted to the Sisters' Hospital, had an Alexander operation performed and has not felt well since that time. She says she has always been regular with her menstruation excepting when pregnant, and until she was operated on. Since then she has felt tired, has had backache, headache, nausea and has lost in weight. She has not menstruated for the last three months. She called her doctor who made an examination per vaginam and made pressure over the abdomen, after which she was obliged to go to bed, became very weak, had severe pain, and showed signs of internal hemorrhages. She was brought to the hospital the next day, twenty-four hours after the examination was made. When admitted she was pulseless, cyanosed and in extremis. On opening the abdomen there was a great quantity of blood and clots, there was a tear in the right cornu of uterus one inch long extending down and posterior to the tube; right tube and ligament clamped and removed. Immediately the abdominal cavity was filled with normal salt solution, while hypodermoclysis, stimulation, and the like, were instituted. A fetus two and a half inches long was found in the pelvic cavity. Patient nearly collapsed on the table. Recovery uneventful.

DECEMBER 7, 1904.

*Fibroid of left ovary—woman pregnant five months. Operation; subsequent delivery at full term.*

Mrs. S., age 40; married two years; gives a clear bill of health in reference to her physical, nervous, and menstrual condition. She had no children. Twenty-four hours before I saw her she was seized with sharp colicy pains in the abdomen, nausea and vomiting. Examination shows a uterus extending nearly to the umbilicus. There was a tumor in the left iliac region about the size of a child's head and movable. Operation: incision was made over site of tumor, which proved to be a fibroid of the ovary four by five inches in diameter with the omentum adhered to it, with a knuckle of intestine strangulated. Omentum was freed, tumor removed; recovery uneventful. Case referred to me by Dr. A. Brennan. *Intraabdominal hernia.* The fetus took care of itself, and I delivered this woman at St. Mary's Maternity Hospital of a nine pound baby three and one-half months afterward.

SEPTEMBER 15, 1906.

*Fibroid and Pregnancy.*

Mrs. D., age 30; was taken sick September 15, 1906. She is about four months pregnant. Sickness began with vomiting, pain in abdomen and unable to pass feces or gas from the bowels. I was called to attend this case in the country, and ordered patient removed to a hospital 15 miles distant, where she was taken on a cot. Diagnosis was obstruction of the bowels due to subperitoneal fibroid. Patient was nearly in a state of collapse. No time was lost in the preparation of the patient; abdomen was opened and a tumor six inches long by three inches thick; pedicle one inch long, with loop of intestine encircling the same was discovered. Tumor was removed and intestine freed; bowels were greatly congested. Patient was about in three weeks. Dr. Charles Banta assisted me. No bad symptoms intervened between the time of operation and delivery. Nurse, Miss N. Glynn, reported the history of labor which began at 3.30 P. M. February 12, and terminated 2:30 A. M. February 13, 1907. Head presentation L, O, A; weight of child eight pounds. Labor normal. Woman was delivered of another child December 25, 1908.

*Interstitial Pregnancy.*

MARCH 1, 1909.

Miss —. Age 19. Never pregnant. Past history negative. Gives no history of being exposed. Only history is that she had been confined to bed for two weeks with pain in the side, says she has always been regular with her menstrual condition and that her menstruation began at the age of 14. Vomiting, bloated, constipated and unable to walk. Examination shows a mass on the right side about four inches long and she experiences great pain upon examination. Operation shows a tube with four inches of ileum attached to serosa. Bowel freed and tube removed. There was a fetus an inch and a half long, partly in the tube and partly in the cornu of the uterus. I cureted from the cornu into the uterine cavity; removed a wedge-shaped portion in the uterus and closed the aperture. Recovery uneventful. This is a true case of interstitial pregnancy without rupture and with operation.

*Fibroid of uterus, and extrauterine pregnancy.*

JANUARY 11, 1905.

Mrs. A. Age 40; nothing out of the ordinary in the previous history; apparently healthy. One child age 15. Regular in her menstruation as regards time, quantity and causes. One month before admitted to hospital was seen by Dr. Pierce Candee, the family physician, who upon making an examination discovered the presence of a fibroid. Patient was prepared for operation; fibroid interstitial; size about 6 in. x 7 in. Growing from the left tube was a tumor that contained a fetus 1½ in. long; hysterectomy. Patient made a good recovery and left the hospital in four weeks.

*Primipara—The necessity of quick operation.*

FEBRUARY 7, 1909.

Mrs. M Age 20, and, by the way, this is a little woman that I brought into the world. Previous history of no special moment, always healthy. Pregnant eight weeks. As this woman lived on the outskirts of the city was seen by Dr. Hourigan, who diagnosticated extrauterine with rupture and advised immediate operation, but the family would not consent without my opinion. Dr. Hourigan informed me over the telephone that he thought the patient would die before she could reach the hospital, if I were obliged to go and see her. I told the husband to send her immediately to the hospital; had everything in readiness when the patient arrived and operated immediately. The abdomen was filled with blood which spouted two or three inches when it was opened; the right tube was ruptured; a fetus about an inch and a half long was found among the clots. Recovery uneventful. Treatment the same as in all extreme cases.

*Operated through the vagina.*

JANUARY 9, 1906.

Mrs. B., widow, age 34, miscarriages two. Was referred to me by Dr. S. of Angola, to be operated on for tumor. Patient complains of bearing down pain, backache, constipation, and gives no history of missed menstrual period and denies having been exposed. Examination shows a large mass in right iliac fossa, also bulging in the vagina, which feels somewhat soft through vaginal vault. Opening was made from below when a great amount of clots was removed. These clots were semiorganised and in the clots was a fetus 1½ in. long. I simply drained and the patient made a good recovery.

*Multipara; hysterectomy; patient in extremis.*

AUGUST 10, 1907.

Mrs. S., age 36; previous history negative. Number of children 6. Weight 110 pounds. Saw this woman just fifteen minutes before I operated upon her. Could get no history prior to operation. She was in extreme shock, brought to the hospital by Dr.

Callahan. I saw her half an hour after being admitted. On arriving at the hospital they informed me that she was dead. The Sister kindly asked me not to operate. Without any preparation, and also disobeying orders, without even removing the patient from the cart, I opened the abdominal cavity in a quarter of a minute, clamped off the tube, found the abdomen filled with blood from the diaphragm to the pelvis. Then I immediately began filling the abdomen with normal salt solution, leaving the clamp in place. Hypodermoclysis and other stimulants given. Patient seemed to rally. As this was a very thin woman and the uterus very movable, in putting on my clamp I not only clamped the tube but the lower segment of the uterus as well. Under the continual flow of normal salt solution into the abdomen, I did a hysterectomy. Patient put to bed, camphorated oil hypodermatically, and continuous salt solution into the rectum. This woman left the hospital in three weeks and a half having received the ordinary nourishing and stimulating treatment. Even the union of the wound was by first intention.

*Ectopic pregnancy—primipara—subperitoneal fibroids.*

APRIL 13, 1909.

Miss B., age 29, occupation, clerk. History not significant. Was called at 3.00 P. M., by Dr. P., to operate for appendicitis. Condition of patient—abdomen distended, pulse 150, barely perceptible, in extremis. Ordered her sent to hospital immediately as she lived only a short distance from the institution. I operated upon her at 4.30. Time under anesthetic about two minutes, as patient was almost moribund. Blood poured from the abdominal cavity after the incision was made. Right tube had been ruptured. There were also present several multiple fibroids. Immediately upon clamping the tube I filled the abdominal cavity with normal salt solution, which is a custom I follow in nearly all of my patients that are in extremis. Left hospital in two weeks and a half.

Subsequent information. She informed me that she had been exposed and that her last menstruation was not similar to her previous ones. I think on account of the number of fibroids that the abnormal gestation was caused by pressure from the tube, destroying its tone.

*Ectopic pregnancy—obstruction of the bowel. Referred by Dr. D. V. McClure.*

FEBRUARY 2, 1909.

Mrs. B., age 34, number of children, four; no miscarriages; youngest child one year old; had had no serious illness; had always been regular with her menstrual periods till November 1, 1908, when she had menorrhagia, which lasted about three weeks. Menstruated December 20, 1908. Said she had been failing in health since October, 1908. She was admitted to the hospital February 2, 1909, suffering from obstruction of the bowel. There had been no movement of the bowels for six days. Abdomen distended, tender, tympanitis; vomiting; patient very anemic. Enema, syrupus fuscus and water given with no effect. Examination per vaginam showed a soft, bogy mass circumscribed and filling the pelvic cavity. Hemoglobin, 30 per cent; white corpuscles 10,600. Patient prepared for operation. An opening was made through the posterior culdesac when a dark sanguinous fluid escaped. On further examination the bowels and omentum were found adhered together by clots. A suprapubic incision was made and pelvis cleaned of all clots and membrane. The right tube was ruptured about the middle third; from appearances it must have contained a fetus of two months' gestation but which was not found. There were over two quarts of blood in the abdominal cavity. It was necessary to separate and wipe several feet of bowel, so matted were they from adhesions. The uterus was twice its normal size, right tube was removed, but the ovary left in place, as it appeared healthy. Abdominal cavity flushed with normal salt solution. A vaginal drain was inserted; patient placed in Fowler's position and stimulants administered. Patient improved and left the hospital March 18, 1909. She has gained twelve pounds and says she feels well.

*Interstitial Pregnancy.—Case sent by Dr. Dewitt Sherman.*

FEBRUARY 22, 1910.

Mrs. A., age 32, number of children 2, youngest five years, eldest eight years. Abortion 1. Previous history not significant. Present history: woman menstruated last, July, 1909. For the first three months she had all the presumptive signs of pregnancy; being an intelligent woman and having borne children she knew that she was in a pregnant state. She had nausea, vomiting, dizziness, frequent micturition,—in fact she was conscious of her condition, and she understood it perfectly. All the symptoms she had with her previous pregnancies were manifested in her present state up till the third month. Then she began to lose in weight, breasts became flat, abdomen, which appeared bloated, lost its contour, became hollow and eyes sunken. As I had attended this woman before I gave a great deal of credence to her history; nevertheless, I could not from my examination consider her nearly eight months pregnant, although she insisted that she was. Examination showed a mass bulging and large on the right side, there is no colostrum in the breasts; she weighs about 110 pounds; she has not menstruated since July 8, 1909, in fact all the probable signs of pregnancy have disappeared. I diagnosticated the case as extrauterine because there is a sulcus between the uterus and the mass. I ordered her to the hospital where she willingly consented to go, fearing that my examination at her home might produce dire results. I was able to move the mass slightly. I examined the uterus with a sound, the depth of which was six inches. I also dilated the os; next day labor pains came on. The external symptoms did not show a pregnancy of from seven to eight months. When labor pains came on I assisted them by dilatation and under chloroform delivered this specimen which I here offer for inspection.

It appears to me to have been an interstitial pregnancy. You will observe the cord is around the neck of the fetus and it appears to have been dead for about four months. She flowed continually after the expulsion of the fetus until April 20, 1910. At present she is improving, hemorrhage having ceased. This did not rupture externally but I think the uterine end of the tube became distended and the sac escaped into the uterus, where it was expelled as a uterine abortion.

### ECTOPIC PREGNANCY.

Opinion.—All patients that have a history characteristic of ectopic should be sent to a hospital, and no forcible examination should be made unless patient is prepared for the worst. All operations should be done immediately, when rupture occurs.

It is better to close all bleeding points with a ligature than to trust to nature. If any mistakes are made let them be on the safe side by operating immediately. When there is great shock open the abdomen quickly, immediately clamp off the tube, fill the abdominal cavity with normal salt solution, then use intravenous injection of salt solution, hypodermoclysis, stimulants, and the like. Laparatomy can be done in half a minute. Hematocele of long standing can be operated from below, but always be prepared to open from above.

To use a quotation, "the general practitioner should be able, in extreme cases, to open the abdominal cavity, tie off the bleeding point, and thus do a life saving operation."

When there is a suspicion that ectopic pregnancy exists it is a good plan immediately to call counsel.

### FIBROID TUMORS COMPLICATING PREGNANCY.

There is great danger of hemorrhage following labor. Tumors increase in size during pregnancy on account of the increased blood supply. It is believed that the danger is not extremely great for operation during pregnancy; that the presence of the tumor is of more danger than the operation; that if there is a pedicle to the tumor it may cause obstruction of the bowel; there may be pressure on the tube; tubal pregnancy may exist also. In many cases fibroids of the uterus apparently cause no bad symptoms during pregnancy. Operations if possible should be avoided at a time that corresponds with the menstrual period. An operation should always be performed when it is indicated to save the life of a patient.

Number of cases, 78; multiparæ, 48; primaparæ, 30; died, 8; no symptoms—no missed menstrual period, 3; full term, 1; cases operated before rupture, 8; fetus found in abdominal cavity, 12; operated at home, 10; rupture during examination, 1; pyosalpinx with ectopic pregnancy, 8; operated through vagina, 8; fibroid of uterus, extrauterine, 6; obstruction of bowel due to extrauterine pregnancy, 1; tubal abortions, 4; same patient operated a second time for extrauterine pregnancy, 1; interstitial pregnancies, 4; immediate operation done in all cases when diagnosis was made excepting, 1.

NOTE—In one case the woman had no signs of pregnancy—was nursing eleven months baby, was pregnant about six weeks, did not know that she was pregnant, was in extremis—left hospital in three weeks—became pregnant four weeks after operation, delivered of healthy baby, no abdominal hernia, has since been delivered of two babies.

428 PORTER AVENUE.

